# Recent Use of Hyaluronic Acid in Dental Medicine

**DOI:** 10.3390/ma18081863

**Published:** 2025-04-18

**Authors:** Giuseppina Malcangi, Alessio Danilo Inchingolo, Irma Trilli, Laura Ferrante, Lucia Casamassima, Paola Nardelli, Francesco Inchingolo, Andrea Palermo, Marco Severino, Angelo Michele Inchingolo, Gianna Dipalma

**Affiliations:** 1 Department of Interdisciplinary Medicine, University of Bari “Aldo Moro”, 70121 Bari, Italy; giuseppinamalcangi@libero.it (G.M.); alessiodanilo.inchingolo@uniba.it (A.D.I.); irmatrilli@hotmail.com (I.T.); lauraferrante79@virgilio.it (L.F.); lucia.casamassima@uniba.it (L.C.); drnardellipaola@gmail.com (P.N.); angeloinchingolo@gmail.com (A.M.I.); gianna.dipalma@uniba.it (G.D.); 2 Department of Experimental Medicine, University of Salento, 73100 Lecce, Italy; andreapalermo@unisalento.it; 3 Department of Medicine and Surgery, University of Perugia, Severi Square n.1, 06132 Perugia, Italy; marco.severino@unipg.it

**Keywords:** Hyaluronic Acid (HA), periodontal therapy, oral surgery, Temporomandibular Joint (TMJ) disorders, tissue regeneration

## Abstract

This systematic review evaluates the clinical effectiveness of hyaluronic acid (HA) in periodontal therapy, oral surgery, and temporomandibular joint (TMJ) disorders. Background. HA, a biocompatible glycosaminoglycan with anti-inflammatory and regenerative properties, is increasingly used in dentistry to enhance healing, reduce pain, and support periodontal regeneration. However, its efficacy compared to conventional treatments remains debated. Materials and Methods. A systematic search was conducted following PRISMA guidelines across PubMed, Scopus, and Web of Science databases (2015–2025). Twenty-one clinical studies, including randomized controlled trials (RCTs) and case-control studies, were analyzed for outcomes related to pain reduction, tissue regeneration, and functional recovery. HA improved clinical attachment levels, reduced probing depth, and enhanced wound healing in periodontal therapy and oral surgery. It accelerated healing after extractions and frenectomies. However, TMJ disorder studies showed mixed results, with some reporting pain relief and functional improvement, while others found no significant advantage over platelet-rich plasma (PRP) or corticosteroids. Variability in HA formulations and protocols influenced outcomes. HA is a promising adjunct for periodontal therapy and wound healing. However, its role in TMJ treatment remains uncertain. Further RCTs with standardized protocols are needed to determine its optimal clinical application.

## 1. Introduction

**Figure 1:**
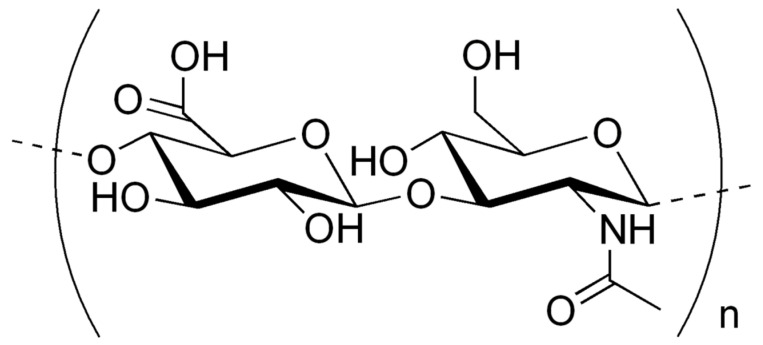
This image depicts the chemical structure of hyaluronic acid.

This article is intended for a broad audience, including dental professionals, researchers, clinicians, and healthcare providers interested in the applications of hyaluronic acid (HA) in dentistry. By summarizing the current evidence and highlighting both clinical and biological mechanisms, this work aims to support evidence-based practice and stimulate further research into the versatile use of HA in oral health and regenerative therapies. HA is a non-sulfated, high molecular weight polysaccharide belonging to the glycosaminoglycan family (Figure 1). HA is present in high percentages in synovial joints, connective tissue, extracellular matrix (ECM) and various body fluids (saliva, synovial fluid, serum, gingival crevicular fluid) [1].

**Figure 2:**
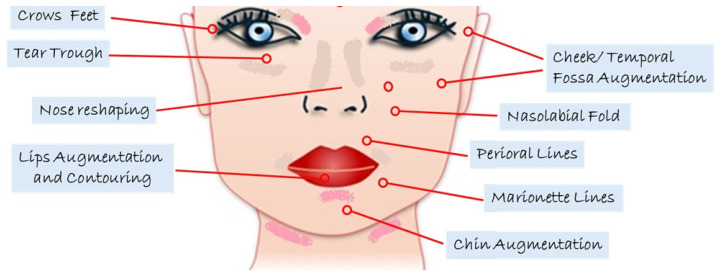
Common indications of HA filler.

In addition to its increasingly common use in aesthetic medicine (Figure 2), for example, in dermal fillers to treat wrinkles and restore facial volume, HA is widely employed in general medicine and dentistry due to its regenerative, anti-inflammatory, and lubricating properties [2].

**Figure 3:**
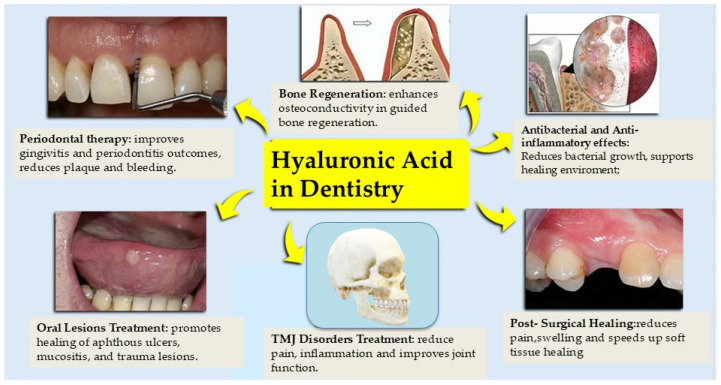
Main uses of HA in dentistry.

HA is biocompatible, immunologically inert and acts as a lubricant by interacting with proteoglycans and other bioactive molecules [3]. The biological activity of hyaluronic acid is largely mediated by its molecular interactions with specific cell surface receptors, primarily CD44, RHAMM (Receptor for HA-Mediated Motility), and TLR2/4. Binding of HA to CD44 initiates signaling cascades that regulate cell adhesion, proliferation, and migration, crucial for wound healing and tissue regeneration. RHAMM, on the other hand, is involved in cell motility and cytoskeletal organization. Additionally, low molecular weight HA fragments can activate Toll-like receptors (TLR2/4), modulating inflammatory responses. These interactions influence ECM dynamics, angiogenesis, and the modulation of oxidative stress, positioning HA as a key player in regenerative and anti-inflammatory processes. HA tissues have been shown in scientific studies to have bacteriostatic, anti-edematous, pro-angiogenic, fungistatic and anti-inflammatory properties [4,5,6,7,8]. In addition to its widely recognised mechanical and hydration properties, recent studies have revealed the role of HA in biochemical and cellular processes under both physiological and pathological conditions, influencing its metabolism [9,10,11,12,13,14,15]. Several HA-interacting membrane receptors such as CD44, RHAMM, HARE and toll-like receptor 4/2 have been found to activate various signalling pathways modulating developmental activity, morphogenesis, tumour activity, cell migration, apoptosis, cell stimulation and inflammation [16,17,18,19,20,21,22,23,24,25]. The biological properties of HA are closely related to its molecular weight. It appears that the use of low and high molecular weight HA has diametrically opposite effects. A high molecular weight HA polymer > 1000 kDa can generate clustering at the cell membrane receptor with anti-apoptotic activity, whereas low molecular weight HA fragments can induce pro-inflammatory effects with increased presence of inflammatory cells and macrophages [26,27,28,29,30,31]. Recognising the multiple biological versatility of HA, its use has also been applied to the soft tissues of the oral cavity [32,33,34,35,36,37,38,39]. Indeed, in recent years it has gained increasing interest as an alternative or complementary therapy to biomaterials, demonstrating benefits in post-surgical (frenectomy, extraction therapy, regenerative surgery) and periodontal therapy, with reduced pain, burning and soft tissue healing times [40,41,42,43,44,45]. The concentration of high molecular weight HA products used ranged from 0.2% to 0.8%. The difference in clinical outcomes may have been influenced by the use of different concentrations, frequency and treatment plan protocol [46,47,48,49,50,51,52]. It has also been shown to be effective in the treatment of aphthous lesions, gingivitis and periodontitis, with significant reductions in bacterial plaque, gingival bleeding and crevicular fluid indices [53]. Compared with the use of other biomaterials used in periodontal regeneration, such as enamel matrix derivatives (EMD) or polynucleotides (PN) or sodium hypochlorite/amino acids, some results suggest that cross-linked hyaluronic acid (xHyA) is proving to be a viable alternative for periodontal regeneration, as well as potentially being cheaper and easier to use, although other studies show its potential as an adjuvant and not necessarily as an alternative to conventional treatments [54,55,56,57]. The stabilisation of blood clots at the site of application would justify HA’s ability to reduce healing time by activating neoangiogenesis, fibroblast migration and reducing the inflammatory response [58,59,60,61,62,63,64,65,66,67]. The use of HA as a scaffold in guided bone regeneration techniques, mixed with autologous bone, alloplastic and xenoplastic materials, has shown increased osteoconductivity [68]. Other research, although promising in clinical trials, has not produced significant results in bone regeneration [69,70,71,72,73,74,75,76,77]. Due to its lubricating and anti-inflammatory properties, HA has been extensively used and studied in the treatment of osteoarthritis (OA) and temporomandibular joint (TMJ) disorders, reducing pain, inflammation and restoring joint function with no adverse events [78,79]. However, intra-articular injections of HA combined with arthrocentesis have been shown to be less effective in relieving pain and opening the mouth than the injection of fibrin-rich plasma, also combined with arthrocentesis [80,81,82]. In contrast, there has been no evidence of benefit from HA injection in TMJ arthroscopy [83,84,85,86,87]. The aim of our research was to evaluate the versatility of HA in dental practice (Table 1) (Figure 3) [88,89,90,91,92,93]. Recent clinical trials have demonstrated significant benefits in a variety of clinical dental problems [94,95,96,97,98,99]. Continuing to explore the various applications of HA and optimising treatment protocols will be critical to maximising clinical benefits and developing innovative therapies to realise the full potential of this biopolymer in dental practice.

- Periodontal Therapy: HA contributes to periodontal regeneration through multiple mechanisms, including its anti-inflammatory and bacteriostatic properties, promotion of fibroblast proliferation, and modulation of cytokine activity. It also plays a critical role in the stabilization of the blood clot and supports extracellular matrix (ECM) remodeling through interaction with CD44 and RHAMM receptors.
- Oral Surgery (e.g., post-extraction healing, frenectomy): HA facilitates wound healing by creating a moist environment, reducing edema, and promoting angiogenesis and epithelial migration. Its viscoelastic nature also helps to maintain tissue space and supports cellular migration.
- TMJ Disorders: Intra-articular injections of HA improve joint lubrication, reduce friction, and modulate inflammatory responses via TLR2/4 and CD44 receptor pathways, which leads to pain relief and enhanced joint mobility.
- Bone Regeneration and Grafting: In bone regeneration, HA serves as a scaffold that improves osteoconductivity, promotes mesenchymal stem cell adhesion and differentiation, and enhances neovascularization—key elements in bone tissue remodeling.
- Oral Lesions (e.g., aphthous ulcers, mucositis): HA accelerates epithelial repair and reduces oxidative stress through antioxidant mechanisms and the downregulation of inflammatory mediators, thereby relieving pain and enhancing mucosal healing.

## 2. Materials and Methods

### 2.1. Search Strategy

This systematic review was conducted in accordance with the PRISMA (Preferred Reporting Items for Systematic Reviews and Meta-Analyses) guidelines and was registered in the International Prospective Register of Systematic Reviews (PROSPERO) under the ID: 1008389. A comprehensive literature search was performed using three electronic databases—PubMed, Web of Science, and Scopus—to identify relevant studies published between 2015 and 2025. The search strategy was developed by combining terms pertinent to the study’s objectives, specifically using the Boolean operators “hyaluronic acid” AND “oral health”.

### 2.2. Eligibility Criteria

Studies were included if they met the following criteria: written in English, available as open access, conducted in vivo or in human subjects, designed as case-control studies, cohort studies, or randomized controlled trials (RCTs), published within the last decade, and focused on adult populations.

These criteria were selected to ensure the inclusion of high-quality, clinically relevant evidence applicable to current dental practice. Randomized controlled trials and observational studies in humans provide stronger levels of evidence for evaluating clinical outcomes and treatment efficacy. Restricting to the past ten years allowed for the inclusion of up-to-date research reflecting current clinical protocols, materials, and therapeutic strategies.

Exclusion criteria encompassed review articles, case reports or series, letters to the editor, studies involving animal models, and in vitro investigations, as these typically provide preliminary or lower-level evidence not directly translatable to clinical decision-making.

All records were independently screened by reviewer pairs, and disagreements were resolved through discussion or third-party consultation.

### 2.3. PICo Framework and Research Question

The research question was formulated using the PICo (Population, Interest, Context) framework to ensure a structured and focused review process.

- Population (P): Patients undergoing treatment for oral and maxillofacial conditions, including temporomandibular joint (TMJ) disorders, periodontal disease, and oral surgical procedures.
- Interest (I): The use of hyaluronic acid (HA) as a therapeutic agent, either as a standalone treatment or in combination with other interventions, aimed at enhancing outcomes such as pain relief, tissue regeneration, and functional recovery.
- Context (Co): Clinical and surgical settings within dentistry and oral medicine, emphasizing HA’s effectiveness compared to standard treatments or placebo.

The principal objective was to evaluate the effectiveness of HA in improving both clinical and radiographic outcomes in various dental and maxillofacial applications, with particular attention to periodontal therapy, TMJ disorders, and oral surgical procedures.

### 2.4. Data Extraction and Analysis

Data extraction was conducted independently by four reviewers (L.C., L.F., I.T., and P.N.) using a standardized form to collect key study characteristics, including design, sample size, intervention details, comparators, follow-up period, and reported outcomes. Discrepancies in data interpretation were resolved through consensus or third-party adjudication. Extracted data were compiled and analyzed using Microsoft Excel and statistical software when applicable. Descriptive statistics were used to summarize study features, while meta-analytical methods were employed where data homogeneity permitted, allowing for quantitative synthesis of HA’s impact on clinical outcomes.

## 3. Results

### 3.1. Selection and Characteristics of the Study

A total of 1096 publications were identified through online databases: PubMed (n = 358), Scopus (n = 178), and Web of Science (n = 560). No additional studies were identified through manual research. After removing 605 duplicate records, 491 studies remained and were screened by title and abstract.

**Figure 4:**
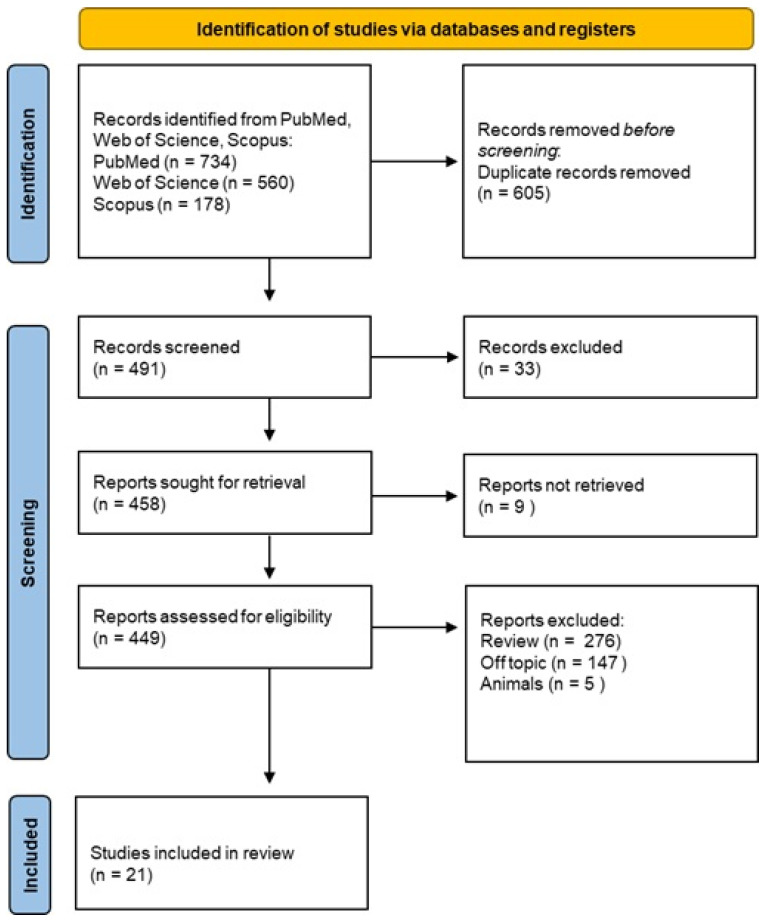
Prisma checklist.

Following a detailed eligibility assessment of the 449 reports, 428 were excluded for not meeting the criteria, leaving 21 studies for qualitative analysis. The selection process and summary of included records are illustrated in Figure 4, while the characteristics of the selected studies are presented in Table 2.

### 3.2. Quality Assessment and Risk of Bias of Included Articles

The risk of bias (Table 3) in the included studies was evaluated using ROBINS-I for non-randomized studies and RoB 2 for randomized controlled trials (RCTs). These tools helped identify potential biases affecting the reliability of results. In RCTs, while most studies used appropriate randomization, some lacked details on allocation concealment, raising concerns about selection bias. Blinding was another issue, as some studies did not clarify whether participants and healthcare providers were blinded, increasing the risk of performance bias, particularly for subjective outcomes like pain perception. Missing data also posed a challenge, with some studies failing to specify how it was handled, weakening the robustness of findings. Furthermore, while most studies relied on validated clinical parameters, some lacked radiographic or biochemical assessments, leading to measurement bias. For non-randomized studies, additional biases emerged, particularly due to inadequate control of confounding factors such as age, disease severity, and concurrent treatments. Some studies also lacked clear inclusion and exclusion criteria, affecting sample representativeness, while others relied on subjective outcome measures without proper blinding, increasing detection bias. Studies were classified into three risk levels (Table 3). Low-risk studies, like Vela et al. [107]. had strong methodologies and transparency. Moderate-risk studies, such as Pilloni et al. [108]. had minor limitations but maintained a solid research structure. High-risk studies, mainly non-randomized, showed issues like selection bias, confounding, and lack of standardized outcome measures, as seen in Tadakamadla et al. [117]. Funnel plot analysis suggested possible publication bias, but the limited number of studies in some subgroups made definitive conclusions difficult. Overall, while most studies provided valuable insights into the efficacy of HA in dentistry, methodological limitations remain. The lack of blinding, unclear handling of missing data, and potential confounding in non-randomized studies highlight the need for well-designed clinical trials with rigorous methodologies to confirm the substance’s efficacy and optimal use.

## 4. Discussion

### 4.1. HA in TemporoTMJ Disorders

HA has been widely studied as a treatment for Osteoarthritis and Temporomandibular Joint disorders due to its potential anti-inflammatory and lubricating properties. However, its effectiveness remains debated, with some studies suggesting it provides significant clinical benefits, while others find no superiority over alternative treatments. Hill et al. (2023) [101] found that FlexPro MD^®^ (containing krill oil, astaxanthin, and HA) significantly improved joint pain and function in mild OA compared to placebo, suggesting its potential as a dietary supplement for OA management. Results showed that FlexPro MD^®^ significantly reduced joint pain, improved physical function, and had fewer adverse events compared to placebo. The study supports the potential of FlexPro MD^®^ as a safe and effective supplement for OA management, though longer-term studies and comparisons with standard OA treatments are needed. Cömert Kiliç et al. (2016) [102] and Castaño-Joaqui et al. (2021) [103] both found that HA did not provide additional benefits over other treatments in TMJ OA. The 2016 study compared PRP vs. HA and found no superiority of PRP, while the 2021 study found that adding HA to TMJ arthroscopy did not improve outcomes significantly. Cömert Kılıç et al. (2021) [104] investigated glucosamine, chondroitin sulfate, and MSM supplementation alongside arthrocentesis with HA injection for TMJ OA. The study found no significant additional benefit from the supplements, aligning with the findings from Cömert Kiliç et al. (2016) [101] and Castaño-Joaqui et al. (2021) [102] studies where HA alone was effective but not superior to alternative or combined treatments. Ozdamar et al. (2017) [104] examined arthrocentesis with and without HA for TMJ internal derangement and found that both treatments reduced pain and improved mouth opening, but HA significantly reduced myeloperoxidase levels, indicating a possible anti-inflammatory effect, though clinical benefits were similar. Yilmaz et al. (2019) [105,106] compared HA injections alone vs. arthrocentesis + HA for TMJ disc displacement and found that both treatments were effective, but arthrocentesis + HA provided superior improvements in chewing efficiency and quality of life, suggesting a combination approach may be more beneficial. Gokçe Kutuk et al. (2019) [107] compared PRP, HA, and corticosteroid injections for TMJ OA and found that PRP was the most effective treatment, surpassing both HA and CS in pain relief and joint function improvement. Overall, while HA demonstrates some potential benefits for OA and TMJ disorders, its effectiveness remains inconclusive, particularly when compared to alternative treatments. Further well-designed, long-term studies are needed to clarify its role in OA and TMJ management and determine the most effective therapeutic approaches.

### 4.2. HA in Periodontal Treatment

The use of adjunctive therapies in periodontal treatment has gained significant attention in recent years. Different biomaterials, including HA, enamel EMD, PN, and sodium hypochlorite/amino acids, have been studied for their potential to enhance periodontal regeneration and improve clinical outcomes. The following discussion integrates and compares several clinical trials that assess the effectiveness of HA-based therapies in combination with other regenerative strategies, highlighting their potential in treating periodontal defects and improving clinical parameters such as PD, CAL and radiographic bone fill. Vela et al. (2024) [108] conducted a randomized, prospective, single-blind clinical study comparing the regenerative effects of xHyA and EMD in the treatment of periodontal intrabony defects. The study involved 60 patients and assessed clinical parameters such as CAL gain, PD reduction, and radiographic improvement over six months. The results demonstrated that both treatments significantly improved periodontal healing, with no statistically significant differences between the two. These findings suggest that xHyA may serve as a viable alternative to EMD for periodontal regeneration, especially considering its potential cost-effectiveness and ease of use. In another study, Pilloni et al. (2023) [56] investigated the clinical effects of a PN and HA-based gel as an adjunct to subgingival re-instrumentation for treating residual periodontal pockets. This randomized, split-mouth clinical trial included 50 patients and compared the effects of the adjunctive treatment (PN + HA) versus re-instrumentation alone. Although both groups showed significant clinical improvements, the test group exhibited slightly better results, particularly in deep residual pockets. However, these differences were not statistically significant, suggesting that while PN + HA may offer additional benefits, it does not substantially outperform re-instrumentation alone. This aligns with findings from other studies that show HA’s potential as an adjunct but not necessarily a superior option compared to conventional treatments. A different approach was explored by Mamajiwala et al. (2021) [109], who evaluated the efficacy of 0.8% HA as an adjunct to OFD for treating periodontal intrabony defects. The study found that after 12 months, the group treated with HA and OFD showed significantly greater CAL gain, PD reduction, and bone defect fill compared to the OFD-alone group. These results suggest that HA gel can significantly enhance periodontal healing and regeneration when used alongside surgical treatment, further supporting its use in combination with surgical interventions. The microbiological effects of adjunctive treatments were also investigated in Ramanauskaite et al. (2024) [110], who assessed the effects of sodium hypochlorite/amino acids and xHyA in non-surgical periodontal treatment. The study showed that the test group receiving adjunctive treatment exhibited significant reductions in periopathogenic bacteria compared to the control group, which only received subgingival debridement. These findings highlight the potential of adjunctive therapies like xHyA to improve microbiological outcomes in periodontal therapy, reinforcing the concept that controlling the bacterial load can play a critical role in enhancing treatment outcomes. In line with this, Benyei et al. (2024) [111] conducted a pilot randomized controlled trial assessing the clinical effects of subgingival instrumentation combined with sodium hypochlorite/amino acid gel and xHyA for treating residual periodontal pockets. This study, conducted over nine months, showed that the test group had better clinical outcomes, including greater PD reduction and CAL gain, compared to the control group. Additionally, gingival recession recovery was more pronounced in the test group. These findings support the use of sodium hypochlorite/amino acid gel and xHyA as adjunctive treatments to improve clinical outcomes, particularly in deep periodontal pockets. However, BOP did not show significant differences between the two groups, suggesting that while the adjunctive treatments can enhance certain clinical parameters, their impact on BOP may be less pronounced.

Finally, Aydinyurt et al. (2020) [112] assessed the biochemical and clinical effects of HA in non-surgical periodontal treatment. Their study compared HA gel and mouth rinse as adjuncts to SRP. While all treatment groups showed significant clinical improvements, the HA gel group exhibited notable changes in biochemical markers such as adenosine deaminase, catalase and glutathione, indicating potential antioxidant and anti-inflammatory effects. However, no significant clinical differences were observed between the HA and control groups. These findings suggest that while HA may influence biochemical markers, its direct clinical benefits may not always translate into significant improvements in clinical parameters in the short term.

In conclusion, the studies suggest that HA, both alone and in combination with other adjunctive treatments, can enhance clinical and microbiological outcomes in periodontal therapy. While it shows promise in regenerative treatments and improving tissue healing, its benefits may not always surpass those of traditional methods such as re-instrumentation or scaling and root planing. Overall, while HA holds potential, further research is necessary to fully define its role and effectiveness in periodontal practice.

### 4.3. HA in Clinical Dental Applications Beyond Periodontics

**Figure 5:**
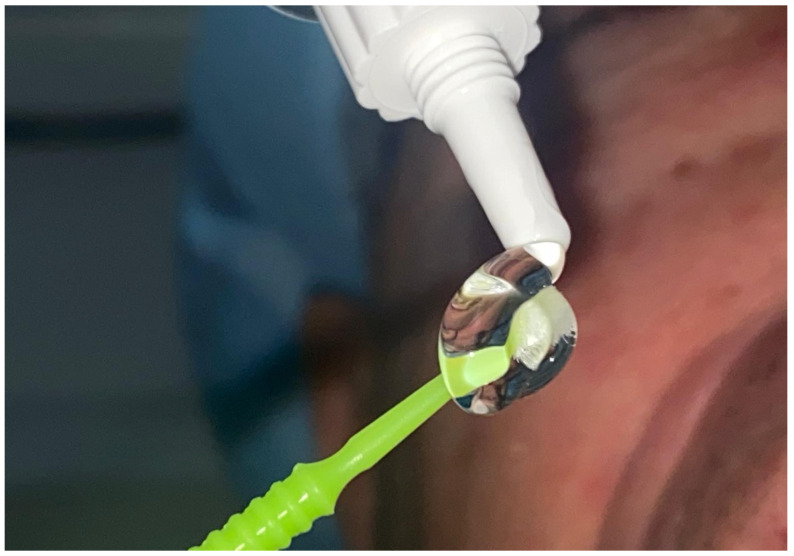
Dental gel based on hyaluronic acid.

**Figure 6:**
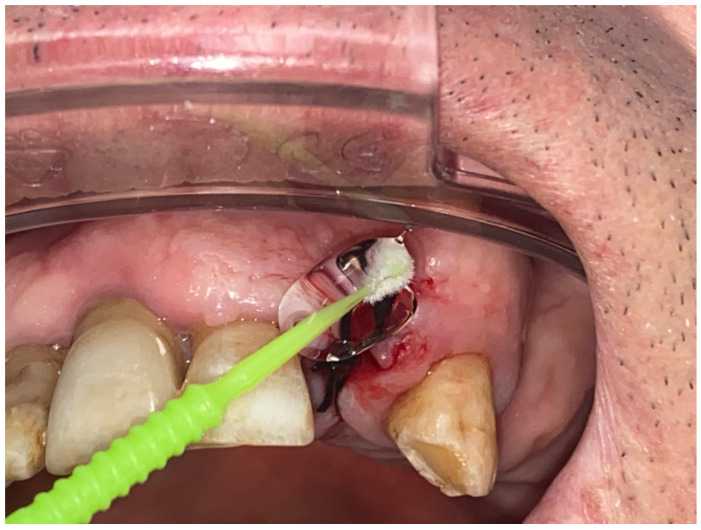
HA used in topic used topically in dentistry after tooth extraction.

In addition to its well-established use in periodontology, hyaluronic acid (HA) has found broader clinical applications across various dental procedures. Its potential to enhance healing, reduce postoperative discomfort, and support both hard and soft tissue regeneration has sparked significant interest among researchers and clinicians alike. This section reviews a series of studies exploring HA’s use in different dental interventions—ranging from tooth extractions and alveolar preservation to soft tissue surgeries and adjunctive therapies—highlighting both the promising results and the limitations observed in clinical outcomes. In recent years, HA has emerged as an increasingly interesting topic in dentistry (Figure 5), thanks to its potential in promoting healing and supporting tissue regeneration. Various studies have sought to evaluate its effectiveness across a range of dental procedures, from tooth extractions to alveolar preservation and even interventions involving soft tissues (Figure 6). For instance, the study by Eeckhout et al. [113] investigated the effectiveness of HA in alveolar preservation. The researchers anticipated that HA would enhance wound healing, but the results revealed no significant differences compared to the control group. Surprisingly, there was actually greater bone resorption in the HA-treated group, raising questions about how and when to use HA in this context. Conversely, the study conducted by Suat Serhan Altintepe Doğan et al. [1] showed much more promising results. Here, the application of a 0.6% HA gel during labial frenectomies in pediatric patients demonstrated a significant improvement in healing and a reduction in postoperative pain, particularly when combined with laser techniques. This suggests that HA can indeed be a helpful adjunct in specific procedures, enhancing patient comfort and accelerating healing.

Further support for the use of HA comes from the study by Elkady et al. [114], which demonstrated that intra-socket application of HA following the extraction of impacted mandibular molars resulted in superior healing of both soft and hard tissues. After two weeks, 100% of patients showed excellent healing, suggesting that HA not only facilitates the healing process but may also accelerate bone regeneration, a crucial aspect following tooth extraction.

In contrast, Ezgi Gurbuz et al. [115] examined the use of a hyaluronic matrix in the context of maxillary sinus augmentation. While there was a significant increase in superficial bone density, other parameters, such as bone volume and trabecular thickness, did not show significant differences. This implies that although HA may have positive effects on bone quality, its overall effectiveness could depend on multiple factors, warranting further research. Shifting to a more innovative approach, Yakout et al. [116] combined HA with photobiomodulation in post-gengivectomy healing. The results were encouraging, showing accelerated healing and significant improvements compared to the control group. This suggests that HA may enhance the effectiveness of other therapies, paving the way for new treatment protocols. From a molecular perspective, the study by Pilloni et al. [117] analyzed HA’s effects on gingival tissue healing, highlighting improvements in ECM remodeling. Although no significant changes in microvascular density were observed, the increased expression of genes associated with collagen remodeling suggests that HA may positively influence the healing process at a cellular level. Another interesting application was examined by Tadakamadla et al. [118], who compared a mouthwash containing cetylpyridinium chloride and HA to chlorhexidine. Both products demonstrated similar effectiveness in plaque control, but HA exhibited fewer side effects, suggesting it could be a viable alternative in preventing gingivitis. Finally, the study by Husseini et al. [119] highlighted the benefits of using xHA in combination with a demineralized bone matrix for alveolar preservation. The results showed a reduction in bone resorption and better integration of new bone, emphasizing the importance of HA in maintaining bone structure following dental extractions.

Overall, the body of evidence indicates that while there are instances where HA may not yield the anticipated results, its applications in specific dental procedures are backed by substantial clinical findings demonstrating enhanced healing, improved tissue quality, and better patient outcomes. The versatility of HA positions it as a valuable tool in modern dentistry. Continuing to explore its various applications and optimizing treatment protocols will be crucial in maximizing the benefits for patients, paving the way for innovative therapies that harness the full potential of this biopolymer in dental practice.

In addition to the promising results described in scientific literature, the clinical use of HA is supported by the availability of numerous commercial formulations specifically designed for oral applications (Table 4). The following table summarizes the main hyaluronic acid-based products used in dentistry, along with their clinical indications and composition.

## 5. Limitations and Future Directions

Although this systematic review highlights encouraging results, several limitations must be acknowledged. The heterogeneity in HA formulations, concentrations, application protocols, and clinical contexts across the included studies impairs the generalizability of outcomes. Most trials had limited follow-up periods and small sample sizes, which may not accurately reflect long-term efficacy. Moreover, objective outcome assessments, such as radiographic and biochemical markers, were inconsistently applied, limiting interpretability regarding tissue healing and regeneration. To improve future research, large-scale, multicenter randomized controlled trials with standardized protocols are necessary. Comparative studies involving HA combined with biomaterials such as EMD, PN, or PRP should be prioritized to determine optimal combinations. Recent studies already point toward the promising synergistic potential of HA with PN and sodium hypochlorite/amino acid gels. Furthermore, advances in biomaterials, including bioengineered or cross-linked forms of HA, have shown enhanced regenerative capacity in preliminary investigations and merit deeper exploration. Inclusion of molecular analyses and validated patient-reported outcome measures will also contribute to a more comprehensive understanding of HA’s efficacy in clinical dental settings [120,121].

## 6. Conclusions

HA has demonstrated significant versatility as a biomaterial in dentistry, with applications in TMJ disorders, periodontal therapy, and oral surgery. In TMJ disorders, HA has shown positive effects in reducing pain and improving joint function, although results vary and often do not exceed those of PRP or physical therapy. In periodontal therapy, HA appears effective in promoting healing and regeneration, particularly when used alongside other treatments like EMD, PN, or OFD, as supported by recent trials. However, it often serves better as an adjunctive aid rather than a standalone treatment. Beyond periodontics, HA has been employed successfully in alveolar preservation, frenectomies, and soft tissue surgeries, contributing to improved healing and patient comfort in selected cases. Nevertheless, inconsistencies in outcomes, such as seen in alveolar preservation studies, highlight the need for more refined treatment protocols. Given the emerging evidence on molecular interactions and material formulations (e.g., cross-linked HA, xHyA), future research should aim to optimize HA-based therapies for specific clinical contexts. A deeper understanding of its biological mechanisms, ideally supported by molecular and patient-centered data, will be crucial for integrating HA more effectively into evidence-based dental practice.

## Figures and Tables

**Table 1:** Applications of HA in Dentistry [100].

Application Area	Description	Key Benefits
Post-surgical healing	Applied after extractions, frenectomy, and gum surgery.	Reduces pain, swelling, and accelerates healing.
Periodontal therapy	Treats gingivitis and periodontitis.	Decreases plaque, bleeding, and pocket depth.
Oral lesion treatment	Used for aphthous ulcers, mucositis, and trauma lesions.	Soothes, protects, and promotes tissue repair.
Bone regeneration	Mixed with bone grafts in Guided Bone Regeneration (GBR).	Enhances osteoconductivity and supports bone healing.
TMJ disorders	Injected into the temporomandibular joint (TMJ).	Relieves pain, reduces inflammation, improves joint function.
Antibacterial effect	Supports healing by reducing bacterial growth.	Lowers infection risk and promotes clean wound environments.

**Table 2:** The table provides an analysis of HA’s effectiveness across these different applications: HA in TMJ (7 articles); HA in Periodontal Treatment (6 articles); other applications of HA (8 articles).

Authors and Years	Type of Study	Aim of the Study and Patients	Materials	Outcomes
Hill W.S. et al. (2023) [101]	Multicenter, Randomized, Double-Blinded, Placebo-Controlled Clinical Trial	To evaluate the efficacy and safety of a Krill Oil, Astaxanthin, and Oral HA Complex on joint health in people with mild OA. 100 patients (57.0 ± 10.28)	FlexPro MD^®^ (Krill Oil, Astaxanthin, and Oral HA; Placebo	Significant reduction in joint pain, improvement in joint function, and better patient-reported joint improvement compared to placebo. Lower incidence of adverse events in the treatment group.
Cömert Kiliç, S. et al. (2016) [102]	Randomized Clinical Trial	To compare the effectiveness of arthrocentesis plus platelet-rich plasma (PRP) versus arthrocentesis plus HA in the treatment of TMJ OA. 31 patients (30.48 ± 13.04)	49 TMJ OA joints in 31 patients; PRP group (arthrocentesis plus PRP injections) vs. HA group (arthrocentesis plus HA injection).	Both treatments led to significant clinical improvements. However, no statistically significant differences were found between the two groups, suggesting that PRP is not superior to HA for TMJ OA treatment. HA injection appears to be more acceptable.
Castaño-Joaqui, O.G. et al. (2021) [103]	Randomized Clinical Trial	To evaluate the effect of HA as an adjunct to TMJ arthroscopy in patients with Wilkes stage-III and stage-IV internal derangement. 51 patients (18–76 years)	51 patients: 25 underwent TMJ arthroscopy alone, 26 received TMJ arthroscopy plus HA injections. Outcomes measured included joint pain, maximum mouth opening and oral health-related quality of life.	Both groups showed improvement in pain, maximum mouth opening, and quality of life, but no statistically significant differences were observed between groups, indicating that HA provided no additional benefit as an adjunct to arthroscopy.
Cömert Kılıç, S. et al. (2021) [104]	Randomized Clinical Trial	To assess whether glucosamine, chondroitin sulfate and methylsulfonylmethane supplementation improves the outcome of TMJ OA management with arthrocentesis plus intraarticular HA injection. 26 patients (28.35 ± 10.85 years)	31 participants with TMJ OA; Control group received a single-session arthrocentesis plus intraarticular HA injection, while the study group received the same treatment followed by 3 months of methylsulfonylmethane supplementation.	Both groups showed significant clinical improvements, but there were no statistically significant differences between them. Methylsulfonylmethane supplementation did not provide additional clinical benefits over HA injection alone.
Ozdamar, S.M. et al. (2017) [105]	Randomized Controlled Clinical Trial	To evaluate the impact of arthrocentesis with and without HA injection on prognosis and synovial fluid myeloperoxidase levels in patients with symptomatic TMJ internal derangement. 24 patients (26.87 ± 7.92)	24 patients with TMJ internal derangement; Group 1 received arthrocentesis with HA, while Group 2 received arthrocentesis with saline solution. Synovial fluid myeloperoxidase levels, pain and maximum mouth opening were measured.	Both groups showed pain reduction and improved maximum mouth opening over time, with no significant differences between them. HA significantly reduced myeloperoxidase levels, suggesting an anti-inflammatory effect, while SS did not.
Yilmaz, O. et al. (2019) [106]	Randomized Clinical Trial	To compare the effectiveness of HA injection alone versus arthrocentesis plus HA injection in treating internal derangements of the TMJ. 90 patients (15–82 years)	90 patients (116 TMJs) with TMJ disc displacement; Divided into groups receiving HA injection alone or arthrocentesis plus HA. Evaluations included pain, mouth opening, chewing efficiency, TMJ sounds, and quality of life over 6 months.	Both treatments improved symptoms, but arthrocentesis plus HA was superior in improving chewing efficiency and quality of life, suggesting that combined treatment is more effective than HA alone.
Gokçe Kutuk, S. et al. (2019) [107]	Randomized Clinical Trial	To compare the clinical effects of intra-articular PRP, HA, and corticosteroid injections in patients with TMJ OA. 60 patients (33.7 ± 10.4 years)	60 TMJ OA patients divided into 3 groups receiving either PRP, HA, or CS injections. Pain, crepitation, loss of function, and loss of strength were evaluated for 3 months.	PRP showed the greatest reduction in TMJ palpation pain compared to HA and CS. PRP also improved joint function and reduced symptoms more effectively, supporting its use as a promising treatment option.
Vela O.C. et al. (2024) [108]	Randomised prospective single-blind clinical study	To compare the regenerative clinical and radiographic effects of xHyA with EMD at six months after regenerative treatment of periodontal intrabony defects. 60 (age no specified)	xHyA, EMD, Periodontal probes, Radiographic imaging software, Various periodontal surgical tools	Both EMD and xHyA produced statistically significant clinical and radiographical improvements after six months when compared with baseline. No significant difference was found between the two treatment modalities.
Pilloni, A (2023) [56]	Randomized, split-mouth clinical trial	To evaluate the clinical effects of PN and HA-based gel as an adjunctive treatment in subgingival re-instrumentation of residual periodontal pockets. 50 patient (age no specified)	PN and HA-based gel, periodontal probes, subgingival instrumentation tools, ultrasonic devices, chlorhexidine mouth rinse.	Both test and control groups showed significant periodontal improvements. The test group (PN + HA gel) exhibited slightly better clinical outcomes, particularly in deep residual pockets, but differences were not statistically significant.
Mamajiwala, A.S (2021) [109]	Randomized controlled clinical trial	To evaluate and compare the clinical and radiographic efficacy of 0.8% HA gel as an adjunct to open flap debridement (OFD) versus OFD alone in the treatment of periodontal intrabony defects. 20 (age no specified)	0.8% HA gel, periodontal probes, cone beam computed tomography (CBCT) imaging, surgical instruments for OFD.	After 12 months, the test group (HA + OFD) showed significantly greater clinical attachment level (CAL) gain, PD reduction, and bone defect fill compared to the control group (OFD alone). HA gel improved clinical and radiographic outcomes in periodontal intrabony defect treatment.
Ramanauskaite E. et al. (2024) [110]	Randomized controlled clinical trial	To investigate the microbiological effects of adjunctive sodium hypochlorite/amino acids and xHyA in non-surgical periodontal treatment. 48 (age no specified)	Sodium hypochlorite/amino acid gel, cross-linked HA gel, subgingival debridement tools, multiplex polymerase chain reaction for bacterial analysis.	The adjunctive application of sodium hypochlorite/amino acids and xHyA led to significant reductions in periopathogenic bacteria after 6 months compared to subgingival debridement alone, supporting its use in periodontal therapy.
Benyei et al., 2024 [111]	Pilot RCT	Assess adjunctive sodium hypochlorite/amino acids + xHyA in residual pockets. 52 patients (mean 58.4 years)	Subgingival instrumentation ± adjuncts, evaluated at 3 and 9 months	Adjuncts improved PD and CAL, especially in deep pockets. No significant bleeding on probing (BOP) difference
Aydinyurt H.S. et al. (2020) [112]	Randomized controlled clinical trial	To evaluate the early-term biochemical and clinical effects of HA as an adjunct to scaling and root planing (SRP) in the treatment of periodontitis. 24 (age no specified)	HA gel, HA mouth rinse, periodontal probes, SRP instruments, spectrophotometric analysis tools for biochemical assessments.	All treatment groups showed significant improvements in clinical parameters and periodontal inflamed surface area after four weeks. Biochemically, groups using HA gel exhibited significant changes in adenosine deaminase, catalase and glutathione levels, suggesting an antioxidant and anti-inflammatory effect of HA. However, no significant clinical differences were observed between the groups.
Eeckhout et al., 2022 [113]	RCT	Evaluate HA gel in alveolar ridge preservation. 38 patients (±53 years)	Collagen graft ± 0.8% HA gel, measured wound dimensions and outcomes for 4 months	No improvement in healing; more bone loss with HA. Not recommended for ARP.
Altintepe Doğan et al., 2024 [1]	Randomized clinical study	Assess 0.6% HA effects on healing after frenectomy. 96 patients (8–14 years)	Conventional/diode laser frenectomy ± HA, assessed at baseline, 1 & 3 months	HA + laser improved healing, reduced inflammation, and enhanced comfort
Elkady et al., 2025 [114]	Randomized controlled trial	Evaluate the effect of 0.8% HA gel on healing after mandibular third molar extraction. 30 patients (21–36 years)	15 patients received HA in the socket, 15 did not. Soft tissue healing assessed on days 3, 7, and 14; bone healing evaluated via CBCT at baseline and after 2 months	HA significantly improved soft tissue healing, increased bone density, and accelerated socket closure compared to controls
Gurbuz et al., 2022 [115]	Case-control study	To assess the effect of hyaluronic matrix on bone microarchitecture after sinus augmentation. 13 patients (33–69 years)	Bilateral maxillary sinus augmentation; test group: hyaluronic matrix + xenograft, control group: xenograft only; MicroCT analysis after 4 months	Hyaluronic matrix increased bone surface density (BS/TV), suggesting improved bone quality, but further research is needed for confirmation.
Yakout et al., 2023 [116]	RCT	Assess HA gel + PBMT on gingivectomy healing 26 patients (18–40 years)	Test: PBMT + 2% HA gel Control: PBMT only Landry’s index at days 3, 7, 14, 21	HA gel improved healing (100% excellent by day 21 vs. 38.5%,
Pilloni et al., 2023 [117]	RCT, split-mouth	Assess HA gel on gingival healing. 8 patients(18–50 years)	HA vs. no treatment; EHS, histological & molecular analysis at 24 h, 1 week	HA improved EHS at 24 h, enhanced ECM remodeling, no effect on angiogenesis
Tadakamadla et al., 2020 [118]	RCT	Compare Complete Blood Count (CBC)-HA mouthrinse with CHX and placebo. 75 patients (age no specified)	21-day, double-blind, three-arm study	CPC-HA and CHX were similarly effective in reducing plaque; CPC-HA did not cause staining.
Husseini et al., 2023 [119]	Randomized split-mouth pilot study	Assess xHyA + DBBM in ridge preservation. 7 patients (mean age 52.65)	Test: DBBM + xHyA, Control: DBBM alone. CBCT at baseline and 4 months. Histology at implant placement.	xHyA reduced bone resorption (*p* = 0.018) but did not affect grafting needs. Improved DBBM integration.

**Table 3:** A summarized overview of the Risk of Bias evaluation for 21 studies, with the assessment covering six key domains.

Authors and Year	Randomization	Blinding	Completeness of Data	Conflict of Interest	Selection Bias	Overall Risk of Bias
Hill W.S. et al. (2023) [101]	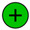	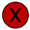	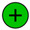	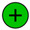	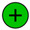	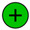
Cömert Kiliç, S. et al. (2016) [101]	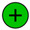	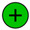	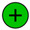	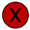	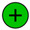	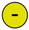
Castaño-Joaqui, O.G. et al. (2021) [102]	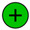	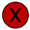	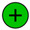	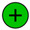	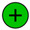	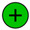
Cömert Kılıç, S. et al. (2021) [103]	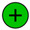	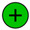	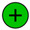	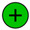	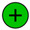	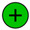
Ozdamar, S.M. et al. (2017) [104]	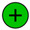	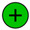	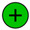	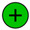	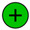	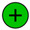
Yilmaz, O. et al. (2019) [105]	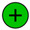	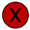	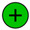	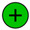	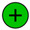	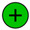
Gokçe Kutuk, S. et al. (2019) [106]	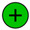	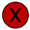	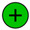	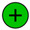	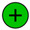	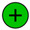
Vela O.C. et al. (2024) [107]	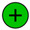	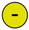	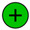	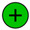	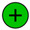	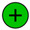
Pilloni, A (2023) [108]	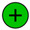	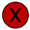	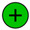	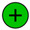	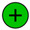	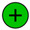
Mamajiwala, A.S (2021) [56]	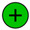	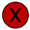	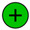	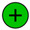	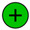	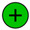
Ramanauskaite E. et al. (2024) [109]	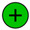	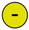	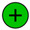	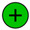	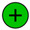	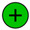
Aydinyurt H.S. et al. (2020) [111]	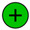	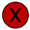	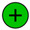	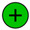	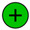	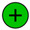
Eeckhout et al. (2022) [112]	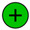	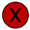	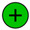	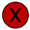	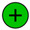	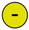
Altintepe Doğan et al. (2024) [1]	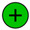	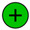	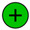	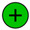	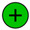	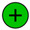
Elkady et al. (2025) [113]	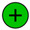	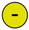	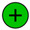	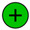	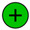	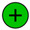
Gurbuz et al. (2022) [114]	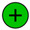	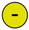	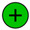	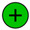	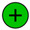	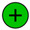
Yakout et al. (2023) [115]	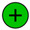	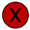	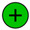	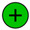	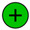	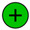
Pilloni et al. (2023) [116]	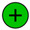	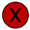	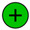	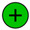	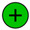	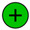
Tadakamadla et al. (2020) [117]	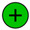	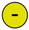	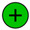	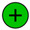	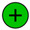	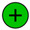
Husseini et al. (2023) [118]	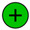	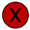	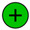	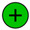	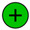	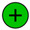
Benyei et al. (2024) [110]	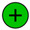	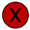	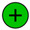	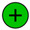	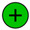	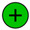
**Domains:**			**Judgement:**			
D1: Bias due to confounding.						
D2: Bias arising from the measurement of the exposure.			Hight	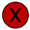		
D3: Bias in the selection of participants in the study (or into the analysis).			Some Concerns	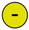		
D4: Bias due to post-exposure interventions.			Low	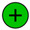		
D5: Bias due to missing data.						
D6: Bias arising from measurement of the outcome.						

**Table 4:** Commercially available hyaluronic acid-based products used in dentistry.

Product Name	Composition	Formulation	Clinical Indications	Application
**Gengigel^®^**	0.2% High-molecular weight HA	Gel, mouth rinse	Gingivitis, periodontitis, post-surgical healing	Topical
**Aftamed^®^**	0.8% HA	Gel, spray	Aphthous ulcers, mucosal lesions	Topical/oral mucosa
**Hyadent BG^®^**	Cross-linked HA	Injectable gel	Periodontal regeneration, surgical defects	Subgingival injection
**Xylmelts^®^**	HA + Xylitol	Oral discs	Xerostomia, mucosal protection	Buccal slow-release
**Bonyf^®^ HA Dental Gel**	HA + natural anti-inflammatory agents	Gel	Periodontal support, mucosal healing	Topical after SRP or surgery
**Hyalograft 3D^®^**	Cross-linked HA + collagen scaffold	Membrane	Bone grafting, regenerative procedures	Surgical placement
**GUM AftaClear^®^**	HA + Aloe vera	Gel, rinse	Oral ulcers, mucosal inflammation	Topical/oral cavity
**Curasept^®^ ADS DNA HA**	HA + DNA + ADS system	Mouth rinse	Periodontal and peri-implant mucositis	Daily rinse
**Amminogam^®^ Gel**	HA + Amino acids (glycine, leucine, lysine, proline)	Gel	Post-surgical healing, mucosal repair, aphthae	Topical (oral mucosa)
**Plakgel Active^®^**	HA + Lactoferrin + Zinc + Aloe vera	Gel	Gingival inflammation, biofilm control	Topical on gingiva or periodontal pockets
**Euclorina Gengiva Gel^®^**	Sodium hypochlorite + HA	Gel	Gingivitis, mucositis, oral hygiene support	Topical (gingival margin)

Note: Product formulations and indications are based on available commercial documentation and scientific literature. Some formulations may vary regionally.

## Data Availability

Not applicable.

## Abbreviations

Abbreviations used in the manuscript in alphabetical order for better readability.

**Abbreviation**	**Full Name**	**Function/Context**
BOP	Bleeding on Probing	Indicator of gingival inflammation in periodontal assessments
CAL	Clinical Attachment Level	Measure of periodontal tissue attachment loss
CBC	Complete Blood Count	A blood test that measures various components of the blood, such as red blood cells, white blood cells, hemoglobin, and platelets.
CBCT	Computed Tomography	Advanced imaging technique used in dentistry and medicine
CD44	Cluster of Differentiation 44	HA receptor involved in cell adhesion, migration, and immune response
ECM	Extracellular Matrix	Structural support network in tissues
EMD	Enamel Matrix Derivatives	Biomaterial used in periodontal regeneration
HA	Hyaluronic Acid	Glycosaminoglycan with regenerative and anti-inflammatory properties
HARE	Hyaluronan Receptor for Endocytosis	Receptor involved in HA clearance and ECM regulation
OA	Osteoarthritis	Degenerative joint disease affecting cartilage
OFD	Open Flap Debridement	Surgical periodontal procedure for deep cleaning and regeneration
PD	Probing Depth	Measure of periodontal pocket depth in periodontal exams
PN	Polynucleotides	Biomolecules used in regenerative medicine
PRP	Platelet-Rich Plasma	Autologous blood-derived product used in tissue regeneration
RHAMM	Receptor for Hyaluronan-Mediated Motility	HA receptor involved in cell motility and wound healing
SRP	Scaling and Root Planing	Non-surgical periodontal therapy for plaque and tartar removal
TMJ	Temporomandibular Joint	Joint connecting the jawbone to the skull
xHyA	Cross-Linked Hyaluronic Acid	Modified HA with enhanced stability for medical applications
